# Doctors Routinely Share Health Data Electronically Under HIPAA, and Sharing With Patients and Patients’ Third-Party Health Apps is Consistent: Interoperability and Privacy Analysis

**DOI:** 10.2196/19818

**Published:** 2020-09-02

**Authors:** Mark Savage, Lucia Clara Savage

**Affiliations:** 1 Center for Digital Health Innovation University of California, San Francisco CA United States; 2 Omada Health San Francisco, CA United States

**Keywords:** digital health, privacy, interoperability, mobile phone, smartphone, electronic health records, EHR, patient access, patient engagement, Health Insurance Portability and Accountability Act, HIPAA, Health Information Technology for Economic and Clinical Health Act, HITECH, covered entity, business associate, protected health information, PHI, digital health applications, apps

## Abstract

Since 2000, federal regulations have affirmed that patients have a right to a complete copy of their health records from their physicians and hospitals. Today, providers across the nation use electronic health records and electronic information exchange for health care, and patients are choosing digital health apps to help them manage their own health and health information. Some doctors and health systems have voiced concern about whether they may transmit a patient’s data upon the patient’s request to the patient or the patient’s health app. This hesitation impedes shared information and care coordination with patients. It impairs patients’ ability to use the state-of-the-art digital health tools they choose to track and manage their health. It undermines the ability of patients’ family caregivers to monitor health and to work remotely to provide care by using the nearly unique capabilities of health apps on people’s smartphones. This paper explains that sharing data electronically with patients and patients’ third-party apps is legally consistent under the Health Insurance Portability and Accountability Act (HIPAA) with routine electronic data sharing with other doctors for treatment or with insurers for reimbursement. The paper explains and illustrates basic principles and scenarios around sharing with patients, including patients’ third-party apps. Doctors routinely and legally share health data electronically under HIPAA whether or not their organizations retain HIPAA responsibility. Sharing with patients and patients’ third-party apps is no different and should be just as routine.

## Introduction

Since 2000, federal regulations have affirmed that patients have a right to a complete copy of their health records from their physicians and hospitals. As the nation transitions to electronic health records (EHRs), electronic information exchange, and health apps that patients choose to help them manage their health and health information, some doctors and health systems have voiced concern about whether they may transmit a patient’s data upon the patient’s request to the patient or the patient’s health app. Physicians worry about their liability under the Health Insurance Portability and Accountability Act of 1996 (HIPAA) if, after transmitting the patient’s data to the patient’s health app, the app then breaches or improperly uses or discloses the data. This hesitation impedes shared information and care coordination with patients. It impairs patients’ ability to use the state-of-the-art digital health tools they choose to track and manage their health. It undermines the ability of patients’ family caregivers to monitor health and to work remotely to provide care, using the nearly unique capabilities of health apps on people’s smartphones.

So, on the road from the doctor’s office to the patient’s third-party app, where are HIPAA’s green lights, yellow lights, and red lights for disclosing patients’ protected health information as patients direct? We explain in detail why it’s a green light all the way, and your patients’ health and care are much the better for it because they can be engaged, informed, and shared decision-makers.

Eleven years after the Health Information Technology for Economic and Clinical Health (HITECH) Act of 2009 [1], clinicians and health systems are increasingly accustomed to transmitting patients’ health data electronically to other doctors, hospitals, labs, pharmacies, and payers, within and outside the sending doctor’s system, for treatment and reimbursement. As of 2015, 96% of hospitals and 78% of physicians had adopted a certified EHR [2]. In 2017, 88% of hospitals and 36% of doctors were sending patients’ health information electronically to care settings and organizations outside the doctors’ health systems [3,4].

Many providers seem less comfortable, however, sharing a patient’s health data electronically with the patient, and even more providers seem hesitant to share a patient’s health data electronically with the patient’s chosen health apps, even though patients have these rights. In 2000, HIPAA’s Privacy Rule required that physicians provide patients with a copy of their health information in physicians’ designated record sets (with some narrow exceptions). In the HITECH Act, Congress requires that physicians who use EHRs give patients electronic copies of their protected health information (PHI) and also requires that physicians who use EHRs follow a direction from a patient to transmit the patient’s PHI electronically to any person, entity, or application the patient chooses [1,5,6]. The Office for Civil Rights has posted an excellent set of frequently asked questions documenting the patient’s right to a copy of the patient’s data and right to have that data sent electronically to any third-party app of the patient’s choice [7-9]. These provisions of the HITECH Act, which apply to EHRs such as those we analyze here, are the law of the land [5].

Sometimes, the provider’s resistance appears to be information blocking [10-14]. But for many, there is concern and uncertainty about transmitting a patient’s data to a health app of unknown security and privacy protection and whether the physician or covered entity may be liable if the patient’s app or its developer subsequently breaches or improperly uses or discloses the data.

This analysis should reassure. We explain that sharing health data electronically with patients and patients’ third-party apps is required and is entirely consistent with physicians’ routine electronic data sharing under HIPAA with other doctors for treatment or with insurers for reimbursement. This paper explains and illustrates basic principles and scenarios around sharing with patients, including patients’ third-party apps. In many common scenarios, physicians’ organizations retain responsibility under HIPAA after sharing, and in others, they do not. In short, doctors routinely share health data electronically under HIPAA, whether or not their organizations retain HIPAA responsibility [15,16]. Sharing with patients and patients’ third-party apps is consistent and should be just as routine, just as banks routinely transmit account information to customers and their smartphone and third-party apps, such as Venmo. To be precise, there is one difference. Doctors’ sharing with others for purposes of treatment, payment, and operations is *permitted* under the Privacy Rule [17], but doctor’s sharing with patients and patients’ third-party apps upon patients’ request is *required* by law [18,19].

While this analysis should reassure, we must note a caveat. This overview serves educational purposes only and does not constitute legal advice. The principles and scenarios that follow illustrate generic situations. In actual situations, analysis depends upon specific facts, circumstances, contractual language, and relationships. However, this summary should help considerably to reduce the uncertainty and friction, and the sources we cite should be well known to providers’ counsel. Moreover, we only address current legal requirements and practices under HIPAA that providers share patients’ health data with their third-party apps upon request. This paper’s scope does not cover how medical ethics and current policy debates treat these requirements. However, for deeper reading on ethics and policy proposals regarding health information disclosure, additional information can be found in [20-24].

References in this paper to terms such as a covered entity, business associate, PHI, disclosure, treatment, operations, use, and breach mean those terms as defined by the HIPAA Privacy Rule in 45 CFR §§160.103, 164.402, and 164.501 (2020). A business associate is a contractor or vendor that a covered entity hires to help that covered entity perform a wide range of health care functions that require that business associate to receive or collect, store, access, use, or disclose PHI. By “affiliated,” we mean covered entities, their business associates (persons or entities that provide a service for or on behalf of a covered entity other than the provision of health care), and their agents. Conversely, by “unaffiliated,” we mean entities or persons that are not legally affiliated under HIPAA, perhaps because they are an independent covered entity or an independent covered entity’s business associate.

## Part 1: Routine Data Sharing Under HIPAA

In general, when a doctor sends a patient’s health data to another provider or system, privately and securely under the circumstances and in the manner allowed by law, the *recipient* is responsible for appropriately securing and handling the PHI after it is received. When a doctor sends a patient’s health data to a doctor within the same health system or to the health system itself, the system’s EHR, or its app, the *affiliated* health system is the recipient and retains responsibility and liability under HIPAA for any subsequent breach or improper disclosure. This should not surprise. The health system is the covered entity under HIPAA and remains responsible for the privacy and security of the health information in its custody and for sending it securely to third parties only when permitted, directed, or authorized by the patient or required by law. Accordingly, the health system must make sure that its business associates, such as its EHR vendors, also abide by these rules.

Conversely, when a doctor sends a patient’s health data to an *unaffiliated* doctor, health system, EHR, or EHR’s app, the sending doctor’s health system does not retain responsibility and liability under HIPAA for any subsequent breach or improper disclosure by that separate covered entity. Instead, the receiving covered entity is responsible under HIPAA for any breach or improper disclosure by itself or its business associates. The same result pertains when the doctor sends the patient’s health data to other unaffiliated covered entities such as payers, laboratories, or pharmacies. This conclusion, too, should not surprise. Organizations expect to be responsible for their own mistakes and expect unaffiliated organizations to be responsible for their mistakes in turn. And HIPAA requires that each has systems in place to avoid mistakes in the first place.

The patient and the patient’s third-party app are just another *unaffiliated* recipient. When a doctor sends a patient’s health data to the patient or to the patient’s third-party app at the patient’s direction and the patient or third-party app subsequently misuses or allows a breach of the data, the doctor’s health system does not retain responsibility and liability under HIPAA for that misuse. The responsibility belongs to the patient or developer of the patient’s third-party app, just as the other unaffiliated recipients described in the preceding paragraph were responsible under HIPAA for their subsequent breach or improper disclosure. Sharing with patients and patients’ third-party apps is no different.

**Figure 1:**
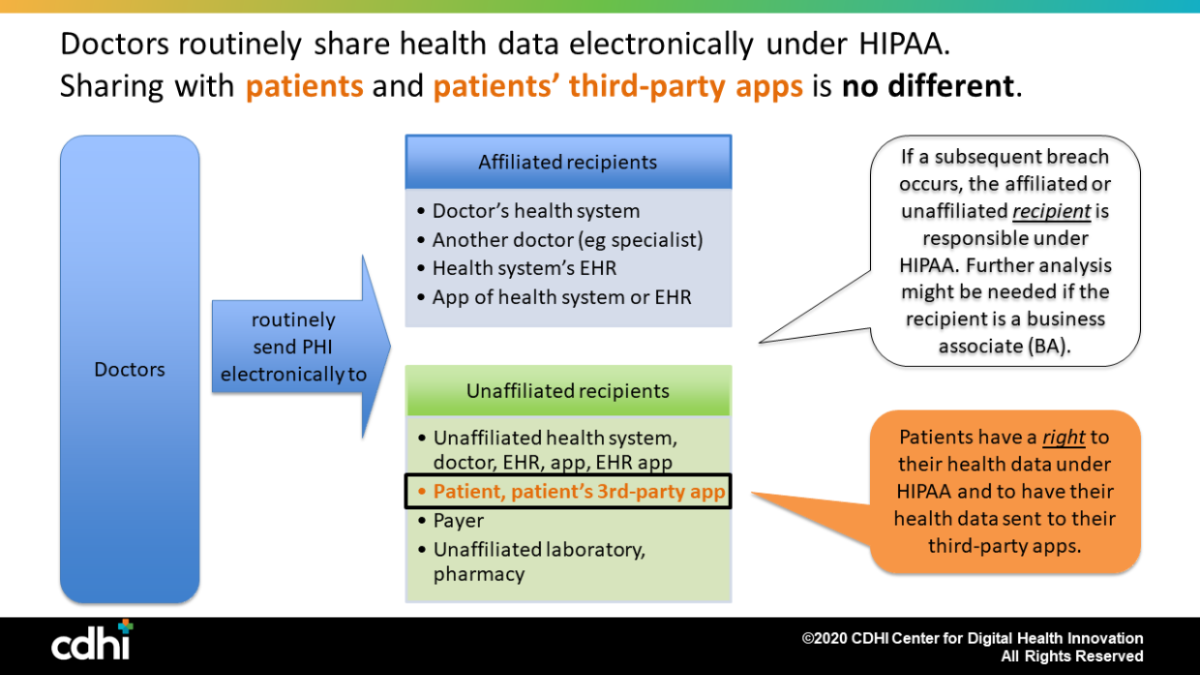
Routine data sharing under the Health Insurance Portability and Accountability Act of 1996 (HIPAA). EHR: electronic health record; PHI: protected health information.

In short, when doctors electronically send protected health information to affiliated recipients or to unaffiliated recipients and the recipient subsequently has a breach or improper disclosure of the data, the *recipient* is liable under HIPAA for its breach or improper disclosure. Doctors should feel just as comfortable with sharing a patient’s health data electronically with the patient herself and her third-party health apps of choice, because the same rule applies (Figure 1).

## Part 2: Patients’ Third-Party Health Apps

Next, we focus on *third-party* apps in more detail, as they seem to be a source of concern or confusion for some doctors and health systems.

In the typical scenario, a patient selects a third-party health app, perhaps from a smartphone or app store, perhaps to help her and her caregivers manage her chronic conditions more effectively. The patient directs her doctor to send a copy of her PHI to the app. The doctor is part of a health system, which is a covered entity under HIPAA, and the covered entity sends the data to the patient’s third-party app as requested.

The relationship between the sending covered entity and the third-party app’s developer, regarding the particular exchange of PHI in question, determines responsibility or liability under HIPAA for a subsequent breach or inappropriate disclosure of the patient’s information.

When the third-party app's developer is an *unaffiliated* covered entity or its business associate or not a covered entity or business associate at all, any “breach” or “improper” use or disclosure under HIPAA would not subject the sending covered entity to liability under HIPAA. For example, when a doctor discloses PHI to an unaffiliated health system’s app for care by a specialist — such as an app for asthma management, heart monitoring, or fertility tracking — the sending doctor and health system are not liable under HIPAA if the recipient app should subsequently breach or improperly disclose the data. The analysis is the same if hospital A discloses to unaffiliated hospital B, to hospital B’s app that hospital B uses to deliver health care, or to an app developed by hospital B’s app developer. In each situation, hospital B has its duties and liability under HIPAA to protect the PHI it received.

When the app's developer is instead *affiliated* with the sending covered entity, the health system generally retains liability under HIPAA for misuses or breaches of PHI by apps it uses or paid to develop. However, further analysis may be necessary, to determine whether the app developer and app were acting on behalf of the sending health system for the particular exchange of PHI in question.

In some rare circumstances, the covered entity may *not* be liable if the app developer’s conduct was outside, or unauthorized by, its contract with the covered entity. Conversely, even though the app developer’s conduct was outside the terms of the business associate agreement (BAA), the covered entity may nevertheless retain some liability for having failed to oversee its app developer or to take action on some activity it should have known was a misuse of the PHI. These are always fact-specific situations, but the following questions illustrate what types of facts are salient: (1) Does the BAA endorse, permit, or not prohibit the business associate’s act in question? (2) Did the sending covered entity know in advance, or should it have known, about the business associate’s act? While the sending health system may have no liability under HIPAA for its business associate’s breach per se, a covered entity still has duties to report and to address a breach of unsecured PHI once discovered (or once it should have been discovered) and may not ignore suspected inappropriate use of data. Liability may adhere for failure to do so. The health system may also have liability if its business associate’s breach entailed noncompliance with HIPAA and the health system knew or should have known about the noncompliance and did not address it. (3) Was the business associate acting as an agent (in the legal sense) of the sending covered entity? Common-law agency generally exists when the sending health system controls or retains authority to control the business associate’s actions with interim direction or instructions as the business associate performs services on behalf of the health system [25].

In some scenarios, therefore, an affiliated app developer may be acting outside the scope of the business associate relationship, and the covered entity may not be liable for the app developer’s extracontractual activity. However, absent facts that support that conclusion, the general principle remains: The health system retains responsibility under HIPAA for its and its business associate’s apps, as usual.

This analysis does not change when the patient directs the health system to send the patient’s PHI to the patient’s health app. The health system’s liability still depends upon the relationship under HIPAA between the sending covered entity and the app's developer. In general, the sending covered entity or its business associate will not be liable under HIPAA for subsequent use or disclosure, unless the app developer is a business associate of and providing services on behalf of the sending covered entity with respect to the disclosure. The patient’s directive to send the PHI is not the salient fact; the salient fact is whether a business associate relationship exists between the health system and the app developer [26].

**Figure 2:**
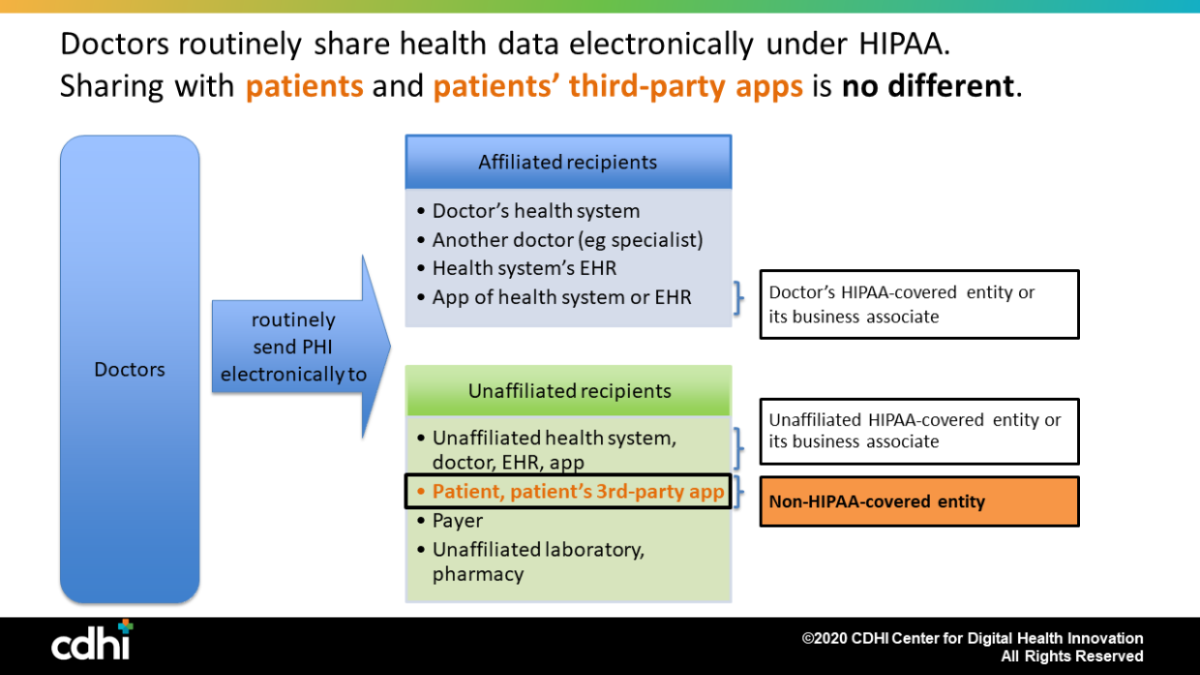
Routine data sharing with Health Insurance Portability and Accountability Act of 1996 (HIPAA)-covered and non-HIPAA–covered entities. EHR: electronic health record; PHI: protected health information.

This introduces an important point that is not reflected in Figure 1. In Figure 1, both the affiliated and unaffiliated apps and app developers were covered by HIPAA and had duties under HIPAA to protect the privacy and security of the patient’s PHI. For example, Omada Health is a covered entity under 45 CFR §160.103 (2020) and is required by law to comply with HIPAA for any PHI it holds. When the apps the patient chooses are *not* HIPAA-covered entities or are not performing their services as a business associate of a covered entity, HIPAA’s requirements and protections do not apply (Figure 2). Here, too, sharing patients’ data with their non-HIPAA–covered apps is no different: Doctors routinely share patients’ health data with entities not covered by HIPAA, such as public health agencies and researchers. Although HIPAA does not apply, other statutes and regulations, such as the Federal Trade Commission’s consumer protection regulations, may apply [24], and this topic has been under active consideration since May 2018 by Congress in various legislative proposals, and more than a half dozen general privacy bills are currently pending. For example, both Senator Wicker’s bill (the Consumer Data Privacy Act) and Senator Cantwell’s bill (the Consumer Online Privacy Rights Act) propose nationwide consumer privacy laws that would require transparent explanations of privacy practices; the right to delete, correct, or port one’s data; choices about collection of certain types of sensitive data, like health data outside HIPAA; and restrictions on use by people other than the original data collector [27,28]. Likewise, while the sending covered entity may have no liability under HIPAA, it may still have liability under other laws or duties, such as medical malpractice or negligence in recommending an app for use.

## Part 3: Twelve Common Scenarios

We illustrate these general principles with 12 common scenarios. In the first set of 3 scenarios, the doctor shares the patient’s data with other parts of the same health system, such as an affiliated specialist, the system’s EHR, or an app the system uses. The second set (scenarios 4-6) covers examples where the doctor instead shares the patient’s data with an unaffiliated health system or covered entity. The third set (scenarios 7-9) focuses on complexities that may arise when the doctor shares the patient’s data with an app affiliated with the doctor’s health system. In these scenarios, the responsibility for breach or improper use depends upon a closer look at the facts and circumstances. Lastly, the fourth set (scenarios 10-12) covers examples where, pursuant to a patient’s direction, the doctor shares the patient’s data with a third-party app the patient independently chose. Together, these scenarios illustrate how sharing data with patients’ third-party apps sits comfortably and consistently within the range of situations where doctors routinely share patients’ data with their own and other health systems.

### Sharing Patients’ Data Within the Doctor’s Health System

As mentioned, we begin with scenarios where doctors are accustomed to sharing a patient’s health data. When doctors share health information with affiliated doctors and apps within the same health system or covered entity, they know that, under HIPAA, the health system remains liable for any breach or improper disclosure by the same health system. When doctors share health information with unaffiliated doctors and apps, then the recipient’s covered entity or business associate is liable under HIPAA. Doctors already routinely share with both.

Scenarios in which the doctors share the patients’ data with other parts of the same health system:

- Scenario 1: A health system’s emergency room doctor shares a patient’s data with the health system’s pulmonologist for the same patient, and after that sharing, there’s a breach or improper disclosure of the data. The health system retains liability where one doctor shares the patient’s data with another doctor within the same health system or covered entity and that receiving doctor improperly uses or discloses the data under HIPAA.
- Scenario 2: A health system’s endocrinologist uses a device or app to share a patient’s data with the health system’s EHR, and the EHR subsequently has a breach or improper disclosure of the data. The health system retains liability where the doctor shares the patient’s data with the health system’s EHR (the EHR vendor being a business associate) and the EHR or EHR vendor improperly uses or discloses the data.
- Scenario 3: A health system’s cardiologist shares a patient’s data from the EHR to a medication management app that the cardiologist has prescribed and the health system developed. The app causes a subsequent breach or improper disclosure of the data. Again, the health system retains liability where the doctor shares the patient’s data with a health app that the doctor prescribed and the health system developed to integrate the data with its EHR for the patient’s care.

### Sharing Patients’ Data With Other Health Systems

We next consider scenarios where doctors know that, after sharing patients’ health data, their health systems no longer retain liability under HIPAA for a subsequent breach or improper disclosure. When doctors share with unaffiliated doctors and EHRs outside their health system and its business associates, they usually know that the receiving covered entity assumes the liability under HIPAA for any subsequent breach or improper disclosure.

Scenarios in which the doctors know that, after sharing patients’ health data, their health systems no longer retain liability under HIPAA for a subsequent breach or improper disclosure:

- Scenario 4: A health system’s doctor shares a patient’s data with a different health system’s doctor for purposes of a second opinion or shares a patient’s billing data with the patient’s health insurer for reimbursement, and the recipient doctor or insurer subsequently has a breach or improper disclosure of the data. The sending health system is not liable when the recipient subsequently has a breach or improperly discloses the patient’s data. Instead, the separate covered entity is responsible under HIPAA for any breach or improper disclosure by itself or its business associates.
- Scenario 5: A health system’s doctor shares a patient’s data with a different health system’s EHR and that EHR subsequently has a breach or improperly discloses the data. Again, the sending health system is not liable when its doctor shares the patient’s data with a separate covered entity’s EHR and that EHR subsequently has a breach or improperly discloses the patient’s data. Instead, the EHR’s vendor is a business associate of the separate covered entity, and the separate covered entity or business associate is responsible under HIPAA for the breach or improper disclosure.
- Scenario 6: A health system’s doctor shares the patient’s data with a personal health record (PHR) app that the patient has chosen and which the doctor’s health system did not develop. The doctor uses the health system’s EHR to transmit the health data. The patient’s PHR app subsequently has a breach or improper disclosure of the data. The sending health system is likewise not liable for the third-party app’s breach, although other laws may make the PHR app liable for its misuse or breach [29].

### Sharing Patients’ Data With Apps Affiliated With the Doctor’s Health System

Next, we consider scenarios where doctors share their patients’ data with affiliated apps, but liability for an app’s subsequent breach or improper disclosure depends upon the facts and circumstances. In each of the scenarios below, where liability lies will depend in part on whether the app developer’s improper use or disclosure fell within or outside the scope of its authority and responsibilities under a BAA with the covered entity, as described in the scenario. Typically, a health system’s legal office will negotiate the BAA with an EHR vendor, an app developer, or a staffing agency, and that negotiation will document the rules to resolve these facts and circumstances.

Scenarios in which the doctors share their patients’ data with affiliated apps, but liability for an app’s subsequent breach or improper disclosure depends upon the facts and circumstances:

- Scenario 7: A health system’s doctor shares a patient’s data with an app that the health system itself or a business associate developed for the system to integrate with its EHR, and the app subsequently has a breach or improper disclosure of the data. Whether the sending health system is liable depends, for example, on whether the health system developed the app or is paying for the app to be available to patients (liable) or a business associate developed the app (may be liable depending on whether the EHR app’s breach or improper disclosure fell within the scope of the app developer’s authority and responsibilities under the BAA).
- Scenario 8: A health system’s doctor shares a patient’s data with the health system’s app. The app’s developer uses the data to push ads about itself to the patient’s friends and family through their social media accounts (which are not regulated by HIPAA). In analyzing liability, even assuming that the app developer was prohibited from this activity, the covered entity might be liable if it knew or should have known that the app developer was using the health information for advertising, as we’ve already discussed.
- Scenario 9: A health system’s doctor shares a patient’s data with the health system’s EHR, and the EHR vendor uses the data to create a research database [30]. Similarly, whether the sending health system is liable depends on whether the EHR’s or EHR vendor’s creation of the research database fell within the scope of the EHR developer’s authority and responsibilities under the BAA. Does the BAA allow or prohibit such a secondary use by the EHR vendor?

### Sharing Patients’ Data With Patients’ Third-Party Apps

The final 3 scenarios concern an app that the patient chooses and the doctor did not sponsor or pay to make available to the patient. The scenarios illustrate, for example, whether the doctor or patient chose the app for treatment. If the patient selected the app, does the analysis change because the doctor subsequently looked at data from the app or even asked the patient to keep sharing the app’s data with the doctor? If the doctor selected the app, but the patient directed the doctor to send the particular data to the app, does the analysis change? We explain why such factors *do not change* the basic principles and results under HIPAA, as already discussed. If the doctor securely sends the data to the patient’s app as directed, the physician is not liable under HIPAA for the app’s conduct after it receives the patient’s data.

Scenarios in which the doctors share their patients’ data with patients’ third-party apps:

- Scenario 10: A patient selects and uses an unaffiliated third-party app such as a fitness tracker, then visits the doctor. The doctor recommends that the patient keep using the app and send the data to the doctor; the patient uploads the data from the fitness tracker to the doctor’s health system through the system’s patient portal. The app subsequently has a breach or improper disclosure of the data. The sending doctor’s health system is not liable for the patient’s app’s breach. The patient’s third-party app developer is not a business associate of the sending covered entity. The doctor’s recommendation and request that the patient show the results to the doctor do not create a business associate relationship between the covered entity and the patient’s third-party app.
- Scenario 11: A patient independently purchases and uses a third-party app such as a diabetes tracker and then visits the doctor (unbeknownst to the patient, the doctor’s health system also uses that app and has a business associate relationship with the app’s developer). After the patient shares the app’s data with the doctor, the doctor recommends that the patient keep using the app and send the data to the doctor. The app subsequently has a breach or improper disclosure of the data. The sending health system is still not liable for the third-party app’s breach or improper disclosure. The patient selected the app, and the app was acting on behalf of and providing services for the patient, not the health system nor its doctor. The fact that a business associate relationship independently exists between the health system and the app’s developer when the doctor prescribes the app for treatment does not create a business associate relationship here, where the patient purchased the app and the app was acting on behalf of the patient.
- Scenario 12: A patient visits a doctor, and the doctor recommends an affiliated app (the doctor’s health system and the app developer have a contract to provide the app and integrate the app’s data in the health system’s EHR). The patient downloads and uses the app, and the app subsequently has a breach or improper disclosure of the data. The app developer is liable under HIPAA for the breach, and the system may be liable if it knew or should have known of the conditions that led to the breach. This is because the app is providing services on behalf of the covered entity, not the patient. It does not matter whether the doctor “recommended” or “prescribed” the app.

Multimedia Appendix 1 introduces some other factors that might or might not cause a doctor uncertainty about whether to transmit the patient’s data and who is liable for a subsequent breach or improper disclosure.

These 12 scenarios all illustrate and bring us back to the common principle in Figure 1. Doctors routinely send protected health information electronically to affiliated recipients (such as doctors in their same health systems and their health systems’ EHRs) and to unaffiliated recipients (such as doctors at different health systems, laboratories, pharmacies, and payers). In both situations, the *recipient* is responsible under HIPAA for its breach or improper disclosure. If the recipient is part of the doctor’s covered entity, then the doctor’s health system retains responsibility under HIPAA. If the recipient is *not* part of the doctor’s covered entity, then the recipient’s covered entity or business associate is liable under HIPAA. Doctors are already routinely sharing with both.

Patients and patients’ third-party apps are no different. Neither patients nor apps they choose independently are recipients affiliated with the doctor or health system, so the doctor’s health system does not retain liability if the patient or the patient’s third-party app subsequently has a breach or improper disclosure of the data.

## Conclusion

Patients have a legal *right* under HIPAA to a copy of their health data and to have their health data sent electronically to a third-party app of their choice. As we have explained, doctors routinely disclose PHI appropriately to other legitimate recipients of that PHI and are not liable under HIPAA for what those recipients do with the PHI. Given these well-established rules and practices, doctors and their health systems should be equally confident in routinely sharing patients’ health data electronically with patients and their third-party apps.

## Appendix

Multimedia Appendix 1 Reference appendix of real-world scenarios with providers sharing health data with patients and their third-party health apps.

## Abbreviations

BAA: business associate agreement
EHR: electronic health record
HIPAA: Health Insurance Portability and Accountability Act of 1996
HITECH: Health Information Technology for Economic and Clinical Health Act of 2009
PHI: protected health information
PHR: personal health record
