# Supplementary material for: Doctors Routinely Share Health Data Electronically Under HIPAA, and Sharing With Patients and Patients’ Third-Party Health Apps is Consistent: Interoperability and Privacy Analysis
Source: J Med Internet Res. 2020 Sep 2;22(9):e19818. doi: 10.2196/19818 (PMC7495255; doi:10.2196/19818)
Supplement: Multimedia Appendix 1 [file jmir_v22i9e19818_app1.pdf]

Liability of Covered Entities, e.g. health systems, for improper use or disclosure by third-party health applications:

Real-world scenarios with providers, patients, third-party health apps

Note for users: This reference begins with core principles that help understand data sharing under the Health Insurance Portability and Accoutability Act (HIPAA) and Health Information Technology for Economic and Clinical Health Act (HITECH), and who is responsibile when there is a subsequent breacher or improper use of the patient's health data. The color-coded charts that follow provide basic scenarios and variations to help illustrate. We group the scenarios into three categories, where the doctor shares the patient's data (1, blue) within the doctor's own health system, (2, gold) with an independent health system, and (3, green) with the patient's third-party app as directed by the patient. The reader can also cross-reference the scenario numbers with the numbered scenarios in the accompanying article for further information.

	Common principles:	Illustrative scenarios
A	Liability under HIPAA depends upon the relationship between the sending health system, i.e. a HIPAA-covered entity (CE), and the app's developer.	Compare scenarios 12, 3, 6, 11
B	When the recipient app's developer is an unaffiliated covered entity (CE) or its business associate (BA), any "improper" (under HIPAA) use or disclosure would not subject the sending CE to liability under HIPAA. The receiving CE or BA has its own separate duties and liability under HIPAA.	Scenarios 6, 11
C	When the app's developer is the sending CE's BA, sending CE's liability for BA's subsequent improper use/disclosure of PHI depends upon the facts and circumstances--in general, whether the BA's improper use/disclosure fell within or outside the scope of its authority and responsibilities under its business associate agreement (BAA) with the health system. For example, (1) does the BAA endorse, permit, or not prohibit the BA's act; (2) did the sending CE know in advance about the BA's act; or (3) was the BA acting as an agent of sending CE—generally, where the sending CE controls or retains authority to control the BA’s actions with interim direction or instructions as BA performs services on behalf of CE. (A BA is a person or entity that creates, receives, maintains, or transmits PHI to perform certain functions or activities on behalf of the CE. Merely specifying the BA’s duties in a contract, or firing or suing the BA for breach, does not create agency.)	Scenarios 3, 7, 8
D	While the sending CE may have no liability under HIPAA for its BA's breach per se, CE still has duties to report and to cure breach of unsecured PHI once discovered or should have been discovered, and may develop liability if it fails to do so. Sending CE may also have liability if its BA's breach entailed noncompliance with HIPAA and CE knew or should have known about the noncompliance and did not address it. However, if the patient directs the CE to send PHI in an unsecure manner, the sending CE is not liable under HIPAA for any breach during transmission and following.	
E	**This analysis does not change when** the patient directs CE to send the PHI. The sending CE's liability **still** depends upon the relationship under HIPAA between sending CE and app's developer. In general, sending CE or its business associate (BA) will not be liable under HIPAA for subsequent use or disclosure, unless the app developer is itself a BA of and providing services on behalf of the covered entity with respect to the disclosure. **The patient’s directive to send the PHI does not determine or change whether a business associate relationship exists between sending CE and app developer.**	Scenarios 6, 8
F	While the CE may have no liability under HIPAA, it may still have liability under other laws or duties, e.g. medical malpractice or negligence in recommending an unaffiliated third-party app for use.	
G	These scenarios illustrate generic situations, and this matrix serves educational purposes only and does not constitute legal advice. In actual situations, analysis depends upon specific facts, circumstances, contractual language, and relationships.	

Scen. No.	Scenarios & sub-scenarios	PHI Source	PHI Recepient vis-à-vis Source	Subsequent event	Sender's liability under HIPAA for subsequent event	Rationale under HIPAA
	**DOCTOR (PRIMARY CARE PHYSICIAN or PCP) SHARES PATIENT'S DATA WITHIN DOCTOR'S OWN HEALTH SYSTEM**					
1	PCP sends PHI to affiliated specialist.	CE	CE	Recepient's improper use or disclosure of PHI	Liable	Covered entities are liable for the actions of their workforce.
	At patient's direction , PCP sends PHI to affiliated specialist.	CE	CE	"	Liable	"
	PCP sends PHI to affiliated specialist's *app.*	CE	CE's BA	"	Could be liable	CE may be liable where BA's improper use/disclosure fell within scope of BA's authority/responsibilities under BAA. See principle C above.
	At patient's direction , PCP sends PHI to affiliated specialist's app.	CE	CE's BA	"	Could be liable	"

(c) 2020 UCSF Center for Digital Health Innovation

Scen. No.	Scenarios & sub-scenarios	PHI Source	PHI Recepient vis-à-vis Source	Subsequent event	Sender's liability under HIPAA for subsequent event	Rationale under HIPAA
2	PCP sends PHI to doctor's EHR.	CE	BA	Recepient's improper use or disclosure of PHI	Could be liable	EHR developer is CE's BA. CE may be liable where BA EHR developer's improper use/disclosure fell within scope of BA's authority/resonsibilites under BAA. See principle C above.
7	PCP sends PHI to PCP EHR's *app.*	CE	BA or sub-BA	"	Could be liable	EHR developer is CE's BA, and app developer may be a BA, too, if developing app for EHR developer on behalf of CE. CE may be liable where BA EHR developer's improper use/disclosure fell within scope of BA's authority/resonsibilites under BAA. See principle C above.
8	*At patient's direction to CE* , PCP sends PHI to EHR's app for CE.	CE	BA or sub-BA	"	Could be liable	"
	PCP's *EHR developer* sends PHI to EHR's app for CE.	BA	BA or sub-BA	"	Could be liable	"
	PCP's EHR developer sends PHI to *EHR developer's app in unaffiliated app store.*	BA	EHR developer's app as separate business	"	Not liable	While EHR developer is BA, EHR developer developed app for its own business purposes and app store, not for CE.
12	Patient visits doctor, doctor recommends *affiliated* app (contract with app developer to provide app and integrate data in EHR), and patient downloads and uses app.	CE	BA	Recepient's improper use or disclosure of PHI	Liable	App provides services on behalf of CE. Irrelevant if "recommendation" or "presciption," or "written instructions."
	Patient visits doctor, doctor recommends affiliated app, provides written *instructions* about what to do, and patient downloads and uses app.	CE	BA	"	Liable	"
	Patient visits doctor, doctor *prescribes* affiliated app, and patient downloads and uses app.	CE	BA	"	Liable	"
3	PCP sends PHI to PCP health system's app.	CE	CE or CE's BA	Recepient's improper use or disclosure of PHI	Could be liable	Liable if CE develops app. If CE's BA develops app, CE may be liable where BA's improper use/disclosure fell within scope of BA's authority/responsibilities under BAA. See principle C above.
	At patient's direction , PCP sends PHI to PCP health system's app.	CE	CE or CE's BA	"	Could be liable	"
	At patient's direction, PCP sends PHI to PCP's *affiliated patient portal.*	CE	CE or CE's BA	"	Could be liable	"

DOCTOR SHARES PATIENT'S DATA WITH AN UNAFFILIATED HEALTH SYSTEM

4	PCP sends PHI to unaffiliated specialist.	CE	Unaffiliated CE	Recepient's improper use or disclosure of PHI	Not liable	Unaffiliated CE is not acting on behalf of sending CE. Unaffiliated receiving CE may be liable, though.
	At patient's direction , PCP sends PHI to unaffiliated specialist.	CE	Unaffiliated CE	"	Not liable	"
5	PCP sends PHI to unaffiliated specialist's *app.*	CE	Unaffiliated CE or unaffiliated CE's BA	"	Not liable	"
	At patient's direction , PCP sends PHI to unaffiliated specialist's app.	CE	Unaffiliated CE or unaffiliated CE's BA	"	Not liable	"

(c) 2020 UCSF Center for Digital Health Innovation

Scen. No.	Scenarios & sub-scenarios	PHI Source	PHI Recepient vis-à-vis Source	Subsequent event	Sender's liability under HIPAA for subsequent event	Rationale under HIPAA
**DOCTOR SHARES PATIENT'S DATA WITH PATIENT'S THIRD-PARTY APP**						
6	At patient's direction, PCP sends PHI to patient's third-party app.	CE	Patient's third-party app	Recepient's improper use or disclosure of PHI	Not liable	Patient's third-party app is not sending CE's BA. See principle E above.
	Patient purchases and uses unaffiliated third-party app (e.g. fitness tracker), and then visits doctor.	CE	Patient's 3rd-party app	Recepient's improper use or disclosure of PHI	Not liable	Patient's third-party app is not the sending CE's BA.
	Patient uses unaffiliated third-party app, visits doctor, and *doctor says, "Keep using it."*	CE	Patient's 3rd-party app	"	Not liable	"
	Patient uses unaffiliated third-party app, visits doctor, and doctor says, "Keep using it and *show me the data."*	CE	Patient's 3rd-party app	"	Not liable	"
10	Patient uses unaffiliated third-party app, visits doctor, and doctor says, "Keep using it and *send me the data."*	CE	Patient's 3rd-party app	"	Not liable	"
	Patient uses unaffiliated third-party app and downloads PHI from doctor's EHR through a patient portal to app.	CE or BA	Patient's 3rd-party app	"	Not liable	"
	Patient uses unaffiliated third-party app, doctor and app developer have interoperability agreement at patient's request to enable secure exchange of PHI between doctor's EHR and app, and patient sends health data from app to EHR and receives test results from EHR to app.	CE or BA	Patient's 3rd-party app	"	Not liable	App not acting on behalf of CE. Interoperability arrangement at patient's request and transmission from app to EHR at patient's request are services to consumer and do not create a BA relationship between CE and app.
	Patient purchases and uses *unaffiliated* third-party app, then visits doctor. Unbeknownst to patient, a separate BA relationship also exists between CE and app developer when system prescribes app.	CE	Patient's 3rd-party app, not BA	Recepient's improper use or disclosure of PHI	Not liable	App is acting on behalf of patient, not CE.
	Patient selects affiliated app, visits doctor, and doctor says, *"Show me the data."*	CE	Patient's 3rd-party app, not BA	"	Not liable	"
11	Patient selects affiliated app, visits doctor, and doctor says, *"Send me the data."*	CE	Patient's 3rd-party app, not BA	"	Not liable	"
	Patient visits doctor, *doctor recommends unaffiliated third-party app* (e.g. fitness tracker), and patient uses app.	CE	Patient's 3rd-party app	Recepient's improper use or disclosure of PHI	Not liable	App is not acting on behalf of CE. Doctor's trust in app does not create a HIPAA relationship with app developer.
	Patient visits doctor, doctor recommends unaffiliated app and says, *"Show me the data."*	CE	Patient's 3rd-party app	"	Not liable	"
	Patient visits doctor, doctor recommends unaffiliated app and says, *"Send me the data."*	CE	Patient's 3rd-party app	"	Not liable	"

(c) 2020 UCSF Center for Digital Health Innovation
